# Targeting RET-rearranged lung cancers with multikinase inhibitors

**DOI:** 10.18632/oncoscience.345

**Published:** 2017-04-14

**Authors:** Joshua K. Sabari, Evan D. Siau, Alexander Drilon

**Affiliations:** Developmetal Therapeutics, Division of Solid Tumor Oncology, Department of Medicine, Memorial Sloan Kettering Cancer Center, New York, NY, 10065, USA; Thoracic Oncology Service, Division of Solid Tumor Oncology, Department of Medicine, Memorial Sloan Kettering Cancer Center, Weill Cornell Medical College, New York, NY, 10065, USA

**Keywords:** non-small cell lung cancer, RET, tyrosine kinase inhibitor, cabozantinib, vandetanib, RXDX-105, LOXO-292, BLU-667

**Figure 1:**
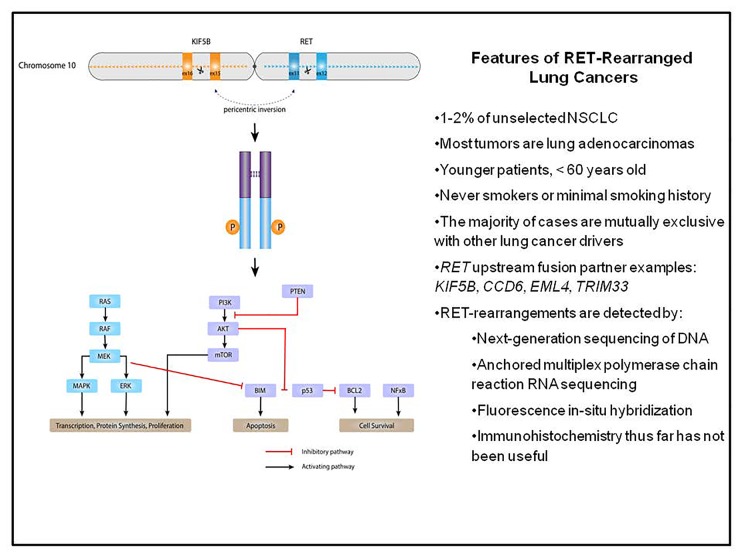
The *KIF5B-RET* rearrangement and clinical features

*RET* rearrangements occur in 1-2% of unselected patients with non-small cell lung cancers (NSCLCs) [1]. The *kinesin family member 5B-RET* (*KIF5B-RET*) rearrangement, the most commonly identified fusion in NSCLCs, was initially described in 2011 in a 33 year-old never smoker with lung adenocarcinoma [2]. The fusion arises from a pericentric inversion in chromosome 10, and the resultant protein activates downstream signaling that drives tumor growth (Figure 1). Alternative upstream *RET* fusion partners include *CCD6, NCOA4, EML4, TRIM33, PARD3, PRKAR1A,* and *ERC1*. *RET* rearrangements are often clonal and are largely mutually exclusive from other oncogenic driver alterations. *RET* rearrangements are commonly found in younger patients (<60 years old), never or former light smokers, and in patients with lung adenocarcinomas, many of which are poorly differentiated [1]. *RET* fusions are actionable targets. *RET*-rearranged lung cancer models respond to multkinase inhibition directed against RET *in vitro* and *in vivo*. Examples of these tyrosine kinase inhibitors are cabozantinib and vandetanib that are approved by the US Food and Drug Administration for the treatment of thyroid cancers.

In 2013, Drilon et al. reported preliminary results of a phase II, single arm, molecularly enriched, study of cabozantinib, a multikinase inhibitor with anti-RET activity, in 3 patients harboring a *RET* rearrangements [3] and updated data after completion of the trial was subsequently published in 2016 [4]. A total of 26 patients were enrolled and treated with cabozantinib and *KIF5B- RET* was the predominant fusion occurring in 62% (16/26) of cases. The study met its primary endpoint, with confirmed partial responses observed in 28% (95% CI 12- 49, n=7/25) of evaluable patients. The most common grade 3/4 treatment related adverse events were elevated lipase, alanine aminotransferase, aspartate aminotransferase, decreased platelets, and hypophosphatemia, and there were no drug-related deaths [4]. This study represented the first foray into targeting *RET* rearrangements in lung cancer and defined a new population of patients that could benefit from further drug development.

Subsequent reports confirmed this activity observed with multikinase inhibition directed against RET. Velcheti et al. next reported the interim results of a phase II study of lenvatinib in *RET*-rearranged lung cancer (NCT01877083) with an overall response rate of 16% (95% CI not reported, n=4/25) [5]. Treatment related adverse events included hypertension, nausea, diarrhea, vomiting, and decreased appetite. More recently, Yoh et al. reported the phase II results of a multicenter Japanese study of vandetanib in patients with advanced *RET*-rearranged lung cancers [6]. Vandetanib displayed clinical antitumor activity with an objective response rate of 53% (9/17) in assessable patients (95% CI 28-77). The most common grade 3/4 treatment-related adverse events were hypertension, diarrhea, rash, dry skin, and QT prolongation. Interestingly, Lee et al. simultaneously reported results from a similar Korean phase II study of vandetanib (NCT01823068), with a response rate that was significantly lower at 17% (95% CI not reported, n=3/18) [7]. Multiple explanations have been proposed to explain the discrepancy in overall response rate between these studies, including differences in patient populations and choice of assay.

Other multi-kinase inhibitors with potential RET activity include sunitinib, sorafenib, alectinib, nintedanib, and ponatinib; however, it is unclear if these drugs are likely to achieve improved responses compared to cabozantinib, vandetanib, and lenvatinib [8]. Given the lower response rates observed in comparison to targeted therapy for *ALK*- and *ROS1*-rearranged lung cancers, the drive to develop novel RET specific inhibitors with improved potency and potentially reduced off target toxicity has led to the development of newer agents that are currently being investigated in the clinical and preclinical setting. A response to RXDX-105, a RET and BRAF inhibitor that relatively spares VEGFR2/KDR and VEGFR1/FLT, has already been reported. A phase I/ Ib study of RXDX-105 with a planned expansion at the recommended phase II dose is ongoing (NCT0187781). Other RET-specific inhibitors in development include LOXO-292 and BLU-667 both potent KDR/VEGF2- sparing RET inhibitors with preclinical specificity for RET and predicted resistant mutants.

Molecular testing remains a critical tool in the oncologist armamentarium. Patients with advanced lung cancer should have routine broad, hybrid capture-based next-generation sequencing of their tumor in an effort to identify actionable genomic alterations. Thus far, there is no reliable IHC assay for the detection of *RET*- rearrangements. When next generation sequencing is not available, it is reasonable to perform fluorescence in situ hybridization (FISH) in patients who are young, never smokers, and have no evidence of other clonal driver alterations. Although less common than other lung cancer drivers, *RET* rearrangements can be successfully targeted, and efficacy may improve with newer agents with a move towards the introduction of RET-specific inhibitors and combination therapy for these patients.
